# A Genome-Wide Association Study for Primary Open Angle Glaucoma and Macular Degeneration Reveals Novel Loci

**DOI:** 10.1371/journal.pone.0058657

**Published:** 2013-03-11

**Authors:** Todd E. Scheetz, John H. Fingert, Kai Wang, Markus H. Kuehn, Kevin L. Knudtson, Wallace L. M. Alward, H. Culver Boldt, Stephen R. Russell, James C. Folk, Thomas L. Casavant, Terry A. Braun, Abbot F. Clark, Edwin M. Stone, Val C. Sheffield

**Affiliations:** 1 Department of Ophthalmology and Visual Sciences, The University of Iowa, Iowa City, Iowa, United States of America; 2 Department of Biomedical Engineering, The University of Iowa, Iowa City, Iowa, United States of America; 3 Center for Bioinformatics and Computational Biology, The University of Iowa, Iowa City, Iowa, United States of America; 4 Department of Biostatistics, The University of Iowa, Iowa City, Iowa, United States of America; 5 DNA Core Facility, The University of Iowa, Iowa City, Iowa, United States of America; 6 Department of Electrical and Computer Engineering, The University of Iowa, Iowa City, Iowa, United States of America; 7 Department of Cell Biology and Anatomy, The University of Iowa, Iowa City, Iowa, United States of America; 8 North Texas Eye Research Institute, University of North Texas Health Sciences Center, Fort Worth, Texas, United States of America; 9 Department of Pediatrics, Howard Hughes Medical Institute, Iowa City, Iowa, United States of America; 10 Howard Hughes Medical Institute, Iowa City, Iowa, United States of America; Duke University, United States of America

## Abstract

Glaucoma and age-related macular degeneration (AMD) are the two leading causes of visual loss in the United States. We utilized a novel study design to perform a genome-wide association for both primary open angle glaucoma (POAG) and AMD. This study design utilized a two-stage process for hypothesis generation and validation, in which each disease cohort was utilized as a control for the other. A total of 400 POAG patients and 400 AMD patients were ascertained and genotyped at 500,000 loci. This study identified a novel association of complement component 7 (C7) to POAG. Additionally, an association of central corneal thickness, a known risk factor for POAG, was found to be associated with ribophorin II (RPN2). Linked monogenic loci for POAG and AMD were also evaluated for evidence of association, none of which were found to be significantly associated. However, several yielded putative associations requiring validation. Our data suggest that POAG is more genetically complex than AMD, with no common risk alleles of large effect.

## Introduction

Age-related macular degeneration (AMD) and glaucoma are leading causes of blindness and visual disability in aging populations. Although these conditions are unrelated, each has a strong genetic component. The glaucomas are a group of diseases that are characterized by injury to the optic nerve and a corresponding pattern of visual loss. These disorders are the second most common cause of irreversible blindness and visual impairment in the United States.[1] The most common form of glaucoma in the United States is primary open angle glaucoma (POAG). Epidemiological studies have shown that POAG is caused, at least in part, by heritable factors. Classic risk factors for glaucoma include advanced age, ethnicity, elevated intraocular pressure, and family history. More recently individuals with relatively thin corneas have been shown to have a three-fold greater risk of developing glaucoma than individuals with normal corneal thickness[2]. Most cases of POAG, and indeed the risk factors of elevated IOP and thin central corneal thickness, are thought to be inherited in a complex fashion[3], [4].

While the genetics of glaucoma are overall complex, a fraction of glaucoma is caused primarily by defects or mutations in single genes. Linkage studies of large families exhibiting autosomal dominant inheritance of glaucoma have mapped the chromosomal locations of multiple different genes capable of causing glaucoma (GLC1A-P; [5]–[17]). The causative genes at six loci have been reported: myocilin (*MYOC*) [18], optineurin (*OPTN*) [19], WD repeat domain 36 (*WDR36*) [14], neurotrophin 4 (*NTF4*) [20], TANK binding kinase 1 (*TBK1*) [17], and ankyrin repeat and SOCS-box containing 10 (*ASB10*) [21]. Mutations in *MYOC* are responsible for ∼4% of cases of POAG. MYOC-associated POAG cases are usually characterized by markedly elevated IOP.[22] Mutations in *OPTN* have been associated with 1–2% of cases of normotensive glaucoma (NTG).[19], [23], [24] The extent to which *WDR36*, *NTF4*, *ASB10* and *TBK1* play a role in the high incidence of POAG is unclear.[14], [20], [21], [25]–[30] Loci and genes have also been reported for primary congenital glaucoma[31]–[36]. In addition, several genetic syndromes are associated with glaucoma.[37], [38]

Genome-wide association studies (GWAS) have begun to identify genes that confer some risk for complex forms of glaucoma (*i.e. caused by the combined actions of multiple genes*). Genes that have been reported to confer an increased risk for POAG or NTG include: *CAV1/2*,[39] *CDKN2B-AS1*,[40]–[43] *ATOH7*,[41], [42], *SIX1*,[41] *TMCO1,* [40] *TLR4*,[44] *SRBD1* and *ELOVL5* [*45*].

Genetic studies of AMD have also identified many important disease-causing genes and risk factors. Linkage analysis and positional cloning studies of pedigrees with Mendelian forms of macular degeneration have identified genes that are likely the primary cause of disease in some patients. For example, a family-based study identified fibulin 5 mutations in members of a large AMD pedigree.[46] GWAS studies have also been highly successful in identifying risk factors of major effect for AMD. The first AMD risk factor to be discovered was a coding sequence variant (Tyr402His) in the complement factor H (CFH) gene.[47]–[50]. Another major AMD risk factor was subsequently identified on chromosome 10. Two genes at this locus (*ARMS2* and *HTRA1*) have been investigated as the source of the AMD risk.[51], [52] Additional AMD risks have been reported for other genes involved in the complement pathway including C2/CFB; C3; C5; C7; SERPING1; CFHR1 +3; CFHR2,4,5; CFI [53]–[60].

The goal of the current study was to use a novel GWAS design to identify additional loci involved in glaucoma and age-related macular degeneration. Importantly, there is no known comorbidity between AMD and glaucoma. We compared the SNP allele and genotype frequencies between two independent cohorts, each with 200 glaucoma patients and 200 AMD patients - a total of 800 patients. Both disease populations were selected to be without a history, clinical signs or symptoms of the other disease. Specifically, the glaucoma patients had no evidence of AMD, and the AMD patients had no evidence of glaucoma. AMD patients were then able to serve as the control (normal) population for the glaucoma cohort and vice versa. This design also incorporated a replication phase. This second 200 versus 200 comparison allowed the validation of the statistically-supported hypotheses generated by the first 200 versus 200 comparison. As all 800 patient samples were evaluated on a genome-wide collection of SNPs (Affymetrix 500k and Mapping 5.0 arrays), a comprehensive comparison of all 400 glaucoma versus 400 AMD patients was also performed in order to maximize our ability to detect associations found through this experiment.

This methodology allowed two simultaneous analyses to be performed, with both cost and time benefits. The disadvantages of this design are that it may be difficult to distinguish the ‘disease phase’ of a given association (i.e., if a given genotypic difference is associated with AMD or glaucoma risk), and that the identification of associations may be confounded by similar disease pathophysiology. Much of the challenge in determining the disease corresponding to a given association was alleviated through the use of expected normal genotypes – for example, the allele and genotype frequencies available as part of HapMap[61] and the Exome Sequencing Project[62]. The design also requires that both cohorts be rigorously assessed to ensure that the AMD patients are free of glaucoma, and vice versa. The number of patients assessed for each phenotype is then equivalent to that of two independent case-control designs.

## Materials and Methods

### Patient Samples

The University of Iowa's Institutional Review Board approved the research study and written informed consent was obtained from study participants. All patients were ascertained in the Department of Ophthalmology and Visual Sciences at the University of Iowa Carver College of Medicine. Blood samples were obtained from study participants and DNA was prepared using a non-organic method as previously described[63].

The primary and validation cohorts within each phenotype category (AMD, glaucoma) were matched for age, disease, gender and sub-phenotypes (dry, unilateral choroidal neovascularization (UCNV), bilateral choroidal neovascularization (BCNV) for AMD; normal tension glaucoma (NTG), high tension glaucoma (HTG) for glaucoma). These cohorts were designed such that the prevalence of disease, sub-phenotype, age and gender were equally represented as shown in Table 1.

**Table 1:** Distribution of patient phenotypes among the primary and validation cohorts.

		AMD			POAG
Phase	Gender	Dry^a^	UCNV^b^	BCNV^c^	
Primary	Female	29	49	48	114
	Male	19	28	27	83
Validation	Female	35	48	45	111
	Male	20	27	25	89

a No evidence for choroidal neovascularization found in either eye.

b Evidence for choroidal neovascularization found in one eye.

c Evidence for choroidal neovascularization found in both eyes.

### Selection of normative data sets

Two normative data sets were utilized in assessing the results of the study. The first was the HapMap[61] CEU population which is comprised of Centre d' Etude du Polymorphisme Humain (CEPH) individuals of Caucasian ethnicity. Population-based allele frequencies for each SNP were provided as part of the annotations available for the Affymetrix genotyping platforms. This population is a representative sample of the general population, and is therefore expected to have the population prevalence of AMD and glaucoma within that population. The second normative population used was a disease-free set of 100 patients drawn from the University of Iowa Ophthalmology clinic. These patients were all over the age of 59 at the time of ascertainment, and had no signs or history of either glaucoma or AMD, as defined below.

### Clinical Criteria – Primary Open Angle Glaucoma

The cohort of glaucoma subjects underwent complete ophthalmologic evaluation including a dilated stereoscopic examination of the optic nerve heads, Goldmann applanation tonometry, gonioscopy, optic nerve head photography, perimetry and slit lamp examination. Visual fields were assessed in using the SITA 24-2 program on the Humphrey Field Analyzer (Humphrey-Zeiss, Dublin, Ca.). Patients that were unable to perform automated perimetry were tested with Goldmann manual kinetic perimetry (Haag-Streit Instruments, Koeniz, Switzerland).

Patients exhibiting excavation of their optic nerve head and associated glaucomatous visual field loss in at least one eye were considered to have glaucoma. Glaucomatous optic nerves were defined as nerves with cup-to-disc ratios of greater than 0.7, thinning of the neural rim, asymmetry of the optic nerve cup-to-disc ratio of >0.2, or photographic documentation of progressive loss of the neural rim. Patients were required to have visual fields of adequate quality for interpretation. For Humphrey visual fields this required a false positive rate, false negative rate and fixation loss rate of less than 33% [64]. Humphrey visual field evidence of glaucoma was based on the Collaborative Normal Tension Glaucoma Treatment Trial criteria[65]. Patients screened using manual kinetic perimetry were required to exhibit depression of the visual field in an arcuate pattern respecting the nasal horizontal meridian. Central corneal thickness was measured using a Pachette 3 ultrasound pachymeter (DGH technology, Inc., Exton, PA). Topical proparicaine was used for anesthesia, the ultrasound probe was placed in apposition to patient corneas and 25 measurements of the corneal thickness were automatically obtained from each eye. The mean of thickness measurements obtained from both eyes was used as the central corneal thickness for each subject.

The medical records of glaucoma patients were reviewed for evidence of macular degeneration. Subjects were excluded from the glaucoma cohort if: 1) they had a prior diagnosis of macular degeneration; 2) they take oral medications for macular degeneration (i.e. AREDS vitamins); or 3) they have a history of treatment with anti-VEGF therapies.

### Clinical Criteria - AMD

Candidates for this project were selected from a pool of patients diagnosed with age-related macular degeneration (AMD) by a faculty ophthalmologist at the University of Iowa. Their charts and photofiles were reviewed by a retinal expert with extensive experience in AMD and AMD trials. For inclusion in this study, a patient had to have either Category 3 or 4 AMD as defined by the Age-Related Treatment Trial in both eyes[66], [67]. For an eye to be classified as Category 3 it must have at least one large (≥125µ) druse, or enough intermediate size (63–125µ) drusen that when the area occupied would be at least 0.5 disc area. A Category 4 eye has advanced AMD defined as geographic atrophy of the retinal pigment epithelium (RPE) in the center of the fovea or choroidal neovascularization. Geographic atrophy of the RPE is defined in the AREDS as the presence of at least two of the three following characteristics: a circular area, sharply defined margins, and visible choroidal vessels. Signs of choroidal neovascularization include elevation of the retinal pigment epithelium, subretinal hemorrhage or fibrosis, serous retinal detachment, hard exudation and leakage of new vessels on fluorescein angiography. If a patient had Category 4 AMD in both eyes, at least one eye had to have at least one large drusen or a 0.5 disc area of intermediate drusen when added together.

Patients with evidence of myopic degeneration, chorioretinal scars in the macula, angioid streaks, or diabetic retinopathy consisting of more than five microaneursyms or hemorrhages were excluded from the study. The few patients who had equivocal findings, no photos, or poor quality photos that could not be evaluated were excluded from the study.

Patients were subdivided into groups based on past or present evidence of CNV up to and including their last follow-up examination. Patients with CNV in both eyes were placed in group one. Patients with CNV in only one eye were placed into group two. Patients with no CNV and were age 70 or older were placed into group three.

The AMD patients were rigorously evaluated for glaucoma to ensure that they could serve as a glaucoma depleted control group. AMD patients were examined and medical records were reviewed by an ophthalmologist that judged them to be free of glaucoma based on the following criteria: 1) history of medication to lower their intraocular pressure; 2) history of any type of glaucoma or status as a glaucoma suspect; 3) cup to disc ratio in either eye of 0.5 or greater unless they had been evaluated by the glaucoma service at UIHC and deemed not to have glaucoma; and 4) intraocular pressure of 25 mm Hg or higher. Patients with an intraocular pressure of 22–24 mm Hg in either eye were included only if there were at least three other measurements that were below this mark.

### SNP Array Genotyping

Genome-wide SNP genotypes were obtained for all samples with the Affymetrix 500 K set and Mapping 5.0 arrays (Affymetrix Inc., Santa Clara, CA) using the manufacturer's recommended protocols. The BRLMM[68] and BRLMM-P[69] algorithms within the Affymetrix Power Tools suite[70] were used to calculate the individual genotypes. Genotypes were called on the 500 K SNP array hybridizations with the BRLMM algorithm as a single group using default parameters. Genotypes were called on the Mapping 5.0 array hybridizations with the BRLMM-P algorithm using default parameters.

### Data management and quality control

SNPs with a call rate less than 85% were eliminated from the analysis. All SNP genotypes were analyzed for Hardy-Weinberg Equilibrium (HWE). A p-value threshold of 0.001 was used to identify SNPs not in HWE. Deviations from HWE may be caused by genuine associations or genotyping errors. Thus all SNPs determined not to be in HWE were manually inspected for evidence of genotyping errors.

### Genetic Analysis

Gender is a known confounder for both glaucoma and AMD. The balanced construction of our populations, however, was designed to minimize gender as a covariate. The Cochran-Armitage test for trend was used to evaluate the genetic variations between the groups of discrete traits (e.g., glaucoma, ocular hypertension) with a correction for population stratification [71] (the 0.1-trimmed variance estimate was used). For continuous traits, genetic association was evaluated through a linear regression in which the dependent variable was the quantitative trait and the predictor variable was the SNP genotype. The possibility of population stratification was corrected for in a similar manner[71]. An uncorrected p-value of 5×10^−7^ was used as the threshold for statistical significance of the associations[72]. P-values less than 1×10^−5^ were classified as suggestive of an association. Only SNPs that were reproducibly associated in both cohorts were considered significant. A cluster of at least three statistically significant (or suggestive) SNPs was required to define an associated (or suggestive) locus, with an inter-SNP distance of at most 200 kb. Associated loci were classified as AMD or POAG using a Fisher's Exact test versus the control populations. To classify the locus, a p-value <0.05 was required for a disease population in both the primary and validation cohorts, with the other disease yielding non-significant p-values (p>0.05).

## Results

### Patient Demographics

Age, gender, and sub-phenotype(s) were evenly distributed within each 200×200 comparison (see Table 1). The absence of inadvertent bias was validated by comparing the distribution of genotypes among the populations using several hundred of the most informative SNPs. These SNPs showed no significant associations, indicating that the hypothesis generating and validating populations had similar ethnic compositions. This was confirmed using the protocol described in by Stokowski et al.[73] using STRUCTURE with a set of genome-wide genomic control SNPs.

### Associated Loci

**Figure 1:**
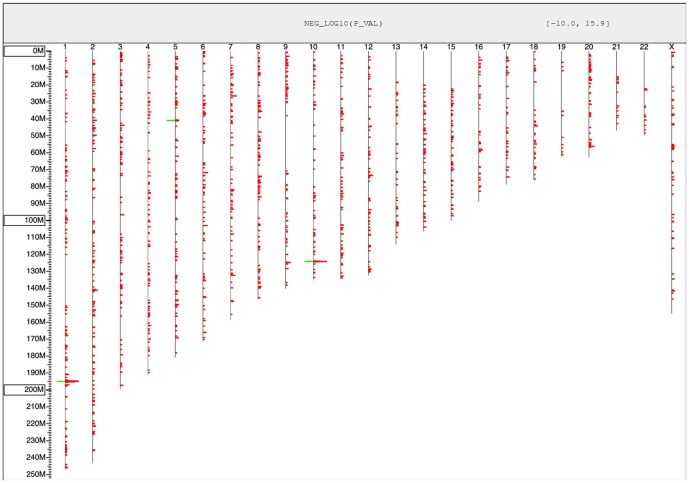
Genome-wide summary of associations. This figure presents the associations identified across all 22 autosomes and the X chromosome. Each vertical line represents a chromosome and the position within that chromosome is given based upon the position along the vertical axis. The length of the red bars represents the strength of the association for individual SNPs, calculated as the negative log_10_ of the association p-value. Only SNPs with p <10^−3^ are shown. The green bars denote the position of the associated SNP clusters, as summarized in Table 2.

Eight hundred patients were genotyped and analyzed in a genome-wide association study design. The resulting associations are summarized in Table 2, ranked by significance. This table lists the associated loci, the p-values from the hypothesis-generating (primary) cohorts, the p-values from the validation cohorts, and the p-values from the combined cohorts. A list of genes within each associated locus is also provided. A graphic depiction of the genome-wide associations is provided in Figure 1. The significance of the associations is represented by red lines to the right of the chromosomes, with stronger associations resulting in longer lines. The associated regions from Table 2 are annotated on this figure in green to the left of the chromosome lines. The associations in Table 2 are classified as either AMD or glaucoma. This assessment was based upon comparison with the expected allele frequencies in Caucasian populations as determined by the two control datasets described previously.

**Table 2:** Summary of associated loci.

Position	Class^a^	SNPs^b^	Best SNP & MAF^c^ (AMD/NL/POAG)	Odds ratio (95% CI)	Joint p-value	p-value (primary)	p-value (replicate)	Genes
**chr1** 194664398-195393272	AMD	31	SNP_A-2171106 (0.16/0.39/0.39)	3.75 (2.91,4.82)	<10^−16^	6.7×10^−13^	1.2×10^−8^	KCNT2, **CFH**, CFHR3, CFHR1P, CFHR4, CFHR2, CFHR5, F13B, ASPM, ZBTB41
**chr10** 124139342-124225345	AMD	7	SNP_A-1841655 (0.45/0.18/0.20)	3.14 (2.49,3.97)	<10^−16^	1.1×10^−7^	3.1×10^−11^	PLEKHA1, **ARMS2**, HTRA1
**chr5** 40965351-40991318	POAG	6	SNP_A-1838573 (0.36/0.36/0.48)	1.65 (1.34,2.03)	5.6×10^−5^	2.2×10^−3^	9.8×10^−3^	**C7**

a Disease classification (AMD or POAG)

b Number of associated SNPs.

c Minor Allele Frequencies (MAFs) presented for AMD, normal controls, and POAG respectively.

As Figure 1 and Table 2 show, the strongest associations are between the two previously reported AMD risk loci on chromosome 1 (complement factor H;[47], [49], [50], [74]) and chromosome 10 (ARMS2; [75], [76]). The strongest association found was that of the Y402H AMD risk-allele in CFH. The reproduction of the two major AMD loci within this analysis served as additional verification of the validity of our study design. Both the CFH and ARMS2 loci are clearly visible with peak associations of p<10^−16^. Also of note based upon previously published results, we carefully evaluated several recently published glaucoma associations [39], [40], [77]–[80]. None of the published associations reached genome-wide significance in our study, however associations to CDKN2B-AS1 and SRBD1 satisfied a gene-level Bonferroni correction with p-values of 0.0002 and 0.0001 respectively. Of the 47 SNPs at the SRBD1 locus only one SNP, rs3213787, produced a significant p-value. This association data provides some support for the previously reported POAG association at the SRBD1 locus. However, the absence of a significant association with other neighboring SNPs suggests our finding at the SRBD1 may be a false positive signal. In contrast, of the 35 SNPs at the CDKN2B-AS1 locus, four produced significant p-values (0.0004, 0.0002, 0.001, 0.0049). This lends additional confidence to the bona fide association of this locus to POAG in our cohort.

Because both populations were selected from the extant clinical population, the relative gender distribution differs slightly between the two disease populations. While the difference is small, we performed a logistic regression as part of the association analysis to address this issue. The resulting p-values were virtually identical to the original analysis, with the expected decrease in significance due to the additional degree of freedom.

### Novel Glaucoma Loci

**Figure 2:**
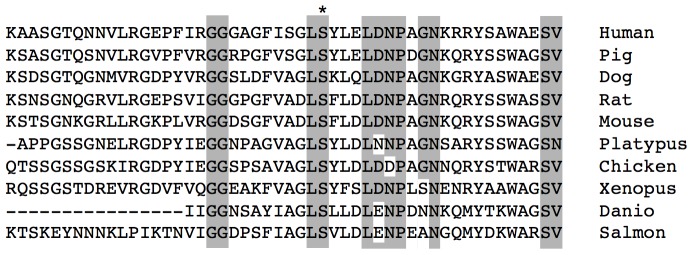
Conservation of the complement component 7 protein. Multiple sequence alignment highlighting the amino acid conservation of the complement component 7 protein (C7) across a broad spectrum of animal species, including mammal, bird, amphibian and fish. The SNP rs1063499 (asterisk) is conserved across these species, suggesting functional conservation of this amino acid.

The genome-wide association analysis identified one glaucoma-associated loci, as shown in Table 2. The peak of the glaucoma association on chromosome 5 occurs within the C7 gene – one of the components of the complement system. This finding is of interest because several complement components have previously been reported as risk-associated factors for AMD. However, based upon the expected allele frequency from HapMap and based upon additional genotyping in a population of normal control individuals, our data strongly suggest that the observed locus is not associated with AMD but represents a novel glaucoma locus. Of the SNPs at the C7 locus showing association with glaucoma, four are found in introns of the gene, and one results in a highly conserved amino acid change (Ser389Thr). While little is known regarding the function of individual protein domains of C7, Ser389 exhibits remarkable conservation in orthologous genes from vertebrate species, suggesting that this region may be functionally important (Figure 2).

### Evaluation of Mendelian POAG intervals

All previously reported loci for monogenic forms of POAG (GLC1A – GLC1P [5]–[16]) were considered *a priori* candidates for association with POAG. Of note, the novel glaucoma-associated regions described above do not overlap this set of published monogenic glaucoma intervals. In this association study, only one of the previously linked loci, GLC1N, exhibited an association (p = 0.00013) to the SNP rs872476. This SNP lies within the THSD4 gene, which is expressed in several ocular tissues including the trabecular meshwork and optic nerve (data not shown).

### Evaluation of Candidate Glaucoma Genes

A set of candidate glaucoma genes was assessed for association in this study. This set of candidate genes consisted of 73 genes localized to the peroxisome, or involved in peroxisome biology as annotated in the Gene Ontology[81], and were selected based upon a recently proposed mechanism for MYOC glaucoma[82]. No associations were found to be both significant and reproducible for these candidates in both cohorts. The best association was found to a SNP (rs2142697) in the hydroxyacid oxidase 1 (HAO1) gene (p = 0.0022). HAO1 was not expressed in any ocular tissues in a high-density gene expression survey of ten ocular tissues (data not shown).

### Central Cornea Thickness

**Figure 3:**
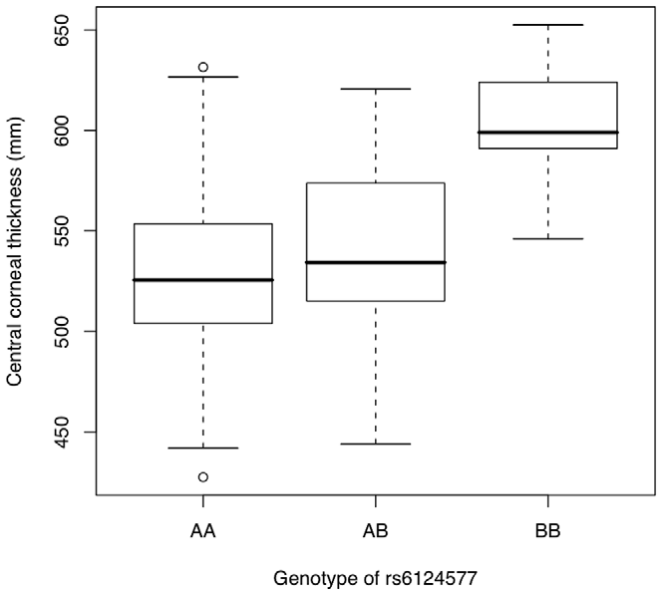
Distribution of central corneal thickness. A boxplot of CCT measurements is presented, demonstrating the differential thickness based upon the observed genotype at rs6124577.

Central cornea thickness (CCT) is one of several quantitative traits reported to be a risk factor for glaucoma. Patients with thin corneas were shown to have a higher prevalence of glaucoma[2]. In this study, CCT data were available for 280 of the 800 patients genotyped. A genome-wide scan was performed to search for genetic variations that modulate corneal thickness. We identified one major QTL on chromosome 20, centered at 35.2 Mb consisting of nine associated SNPs. The distribution of CCT values in the three genotype classes of rs6124577 is shown in Figure 3. This figure shows that AA homozygotes and AB heterozygotes have significantly thinner corneas compared to BB homozygotes. This locus contains the RBL1, C20ORF132 and RPN2 genes, with the strongest association within the RPN2 gene. Although the p-values are not significant at the genome-wide level, this cluster is reproducibly observed in the primary and validation cohorts.

### AMD Loci

This study identified the two previously reported AMD loci CFH[47], [49], [50], [74] and chromosome 10[75] (Table 2). The chromosome 10 locus contains the PLEKHA1, ARMS2 (LOC387715) and HTRA1 genes. There is evidence for each of these genes to be the risk-associated gene[51], [75], [83]. Our analysis found the strongest association to the Arg69Ser variation in ARMS2 reported by Swaroop et al. [76].

A set of previously reported AMD causing or AMD risk associated loci (RetNet.org; [84]) were also evaluated. The analysis of these loci is presented in Table 3. Three of the loci (BSMD, MCDR3 and MCDR4) were associated with AMD in the primary and validation cohorts. The BSMD locus contains a cluster of associated SNPs with a peak p-value of 6.2×10^−5^. The associated SNPs localize within the first intron of the CAMK2A gene, a calcium-dependent serine/threonine kinase. The MCDR3 locus contains a cluster of eight associated SNPs with a peak p-value of 2×10^−4^. This associated locus contains the CCT5 gene, part of the chaperonin containing TCP1 complex, and the first exon of the FAM173B gene. The MCDR4 locus contains three associated SNPs with a peak p-value of 0.002. There are no annotated genes within the MCDR4 associated SNP cluster.

**Table 3:** Evidence for association to linked AMD loci.

Linked Locus	Genomic Position	Peak p-value	Primary p-value	Validation p-value
BCMAD	chr6:45098201-104690621	1.1×10^−6^	8.0×10^−1^	1.1×10^−10^
BSMD	chr5:121308199-155661743	6.2×10^−5^	4.9×10^−3^	5.1×10^−3^
MCDR1	chr6:75845485-99792538	3.4×10^−4^	1.0×10^−4^	8.2×10^−1^
MCDR3	chr5:988510-21094862	2.5×10^−4^	2.4×10^−2^	4.0×10^−3^
MCDR4	chr14:20983113-21023115	2.9×10^−3^	4.1×10^−2^	3.4×10^−2^
MCDR5	chr19:44719578-46244168	3.3×10^−3^	2.2×10^−4^	N/A
MDDC	chr7:22317857-26483126	5.4×10^−8^	6.6×10^−1^	1.2×10^−11^
TEAD1	chr11:12703561-12963122	1.2×10^−2^	4.9×10^−2^	1.2×10^−1^

### AMD associated genes

We also assessed the set of published AMD-causing or risk-associated genes, as well as complement-related genes for evidence of an AMD association. These results are summarized in Table 3. As described above, both CFH and ARMS2 are significantly associated in the genome-wide screen. From the set of AMD causing and risk-associated genes, suggestive and reproducible associations were detected for C2/CFB, C3, HTRA1 and PROM1. The association to C2 and CFB comes from a SNP (rs522162) that is immediately 3′ of CFB. However, given the proximity of C2 and CFB this SNP may be in linkage disequilibrium with a causative variation in either or both genes. Eight additional complement associated genes were identified with suggestive, reproducible AMD associations, specifically ADCY3, CFHR2, CFHR3, CFHR4, CFHR5, F13B, ASPH and CSMD1. It should be noted that the apparent association for CFHR2-5 and F13B may be due to their proximity to CFH.

## Discussion

The design of this study utilized two separate affected populations, glaucoma patients and macular degeneration patients. Each patient population served as the normal control for the other. This study design allowed maximum efficiency in utilization of the genetic data in the assessment of genetic associations. A limitation of this study design is that the diseases to be studied should not share genetic risk factors with each other. Without this constraint, loci contributing to both disorders would likely go undetected. For example, if the Y402H variation in complement factor H was a risk allele for both glaucoma and AMD, the likelihood for identification of this variation as a risk allele for either disorder would be diminished compared to studies using normal controls (patients with neither glaucoma or AMD).

Provided that two diseases do not share risk associations, the study design used here allows the identification of SNPs that are statistically associated to one of the diseases or the other. However, the *disease classification* of each individual association (i.e., in which disease the genetic variant is acting) is not immediately apparent. In such instances, it is possible to utilize the expected allele and genotype frequencies based upon other studies such as the HapMap project[61]. The disease population differing from the ‘normal’ frequency determines the disease classification of the association. It is possible that, in rare instances, a given allele could be a risk factor for one disorder and a protective allele for the second disorder. In such an instance the risk allele would be enriched in one population and depleted in the other (compared to normal controls), providing an increase in statistical power to detect the association. In the current study, the previously reported AMD associations, CFH and ARMS2, were readily detected as AMD associated loci using the glaucoma cohort as the control, with allele frequencies similar to those reported in other studies. These findings validate the use of glaucoma controls for AMD studies, and indicate that CFH and ARMS2 are not involved in glaucoma.

The primary advantage of the two-disease study design is that genotyping costs can be conserved in the initial phase of the study (genotyping of the primary cohorts). This allows hypotheses to be developed for both diseases for a specific population without genotyping an additional normal control cohort. The power to detect association is based upon the size of the two disease cohorts. Hypotheses developed in the initial phase, can then be pursued using a validation cohort. A control cohort can be included for limited genotyping at loci of interest and to clarify the specific disease associations. In this study two control cohorts were utilized – the CEU population from HapMap and a set of normal patients from the same clinic population as the POAG and AMD cohorts. The allele and genotype frequencies from the normal cohorts were in close agreement with each other.

In this study, no single, large risk allele for glaucoma on the order of the CFH AMD risk allele was detected. Therefore it is likely that this disorder is very genetically heterogeneous and that no single locus with a high genotype relative risk (GRR) exists for POAG in the population studied. A key factor in the power to detect associated loci in a GWAS analysis is the genotype relative risk. The GRR reported for CFH and ARMS2 (2.44–5.93 and 3.2–7.9, respectively) contribute substantially to the ability to detect those associations[74], [75]. In contrast to POAG, a single locus on the LOXL1 gene significantly contributes to elevated risk for developing exfoliation syndrome[85], a condition that is highly prevalent in some populations and may lead to secondary glaucoma.

The findings highlight the genetic complexity of glaucoma. Known Mendelian genes account for between three to five percent of glaucoma, with the remainder of disease most likely resulting from a combination of many risk factors, each with a small effect. Previous GWAS reports have identified several glaucoma risk associated genes (*CAV1/2*,[39] *CDKN2B-AS1*,[40]–[43] *ATOH7*,[41], [42], *SIX1*,[41] *TMCO1*,[40] *TLR4*,[44] *SRBD1* and *ELOVL5* [45]). However, none of these risk factors were associated with glaucoma in our study at genome-wide significance. A single gene, SRBD1, was found to be significant under a more permissive multiple hypothesis correction strategy, in which only the eight risk-associated genes were evaluated (p<0.00625). These data underscore key features of the genetic complexity of glaucoma. No genetic risk factors of major effect have been discovered for POAG, and the identified risk factors of smaller effect are more important in some populations than others.

This study is adequately powered to detect genetic effects where additional copies of the disease allele increase the odds of disease by a factor of 2 or larger unless the disease-associated allele is rare. For instance, OR> = 2 and MAF>0.1 (Table 4). For smaller odds ratios, the power is lower unless the disease-associated allele is common, for instance, OR = 1.75 and MAF = 0.4 (Table 4).

**Table 4:** Power to detect genetic effect.

OR	Minor Allele Frequency	Power (α = 5×10^−7^)	Power (α = 1×10^−5^)	Power (α = 5×10^−5^)
1.75	0.10	0.011	0.044	0.104
	0.20	0.149	0.367	0.509
	0.30	0.343	0.625	0.773
	0.40	0.535	0.792	0.865
2.00	0.10	0.042	0.164	0.277
	0.20	0.417	0.665	0.798
	0.30	0.758	0.912	0.960
	0.40	0.892	0.971	0.990
2.50	0.10	0.221	0.480	0.636
	0.20	0.866	0.961	0.986
	0.30	0.984	0.995	0.999
	0.40	0.998	0.999	1.000

OR is the odds ratio of disease risk for genotype Dd versus genotype dd (assumed to be equal to the odds ratio for genotype DD versus Dd). The disease risk for genotype dd is assumed to be 0.001. Power is simulated over 1000 replications.

### Complement factor 7 (C7)

Despite the lack of strongly associated loci for POAG or strongly associated novel loci for AMD, several loci and genes of interest were identified. Of particular interest to the study of glaucoma is the association of C7. C7 has several features that make it an interesting glaucoma candidate. Although C7 is widely expressed, it is highly expressed in optic nerve (data not shown), a tissue of critical relevance to glaucoma. Our data indicate that there are several SNPs within the C7 gene that are significantly associated with glaucoma. Four associated SNPs are located in an intron. An additional SNP encodes an amino acid change (Ser389Thr), which is phylogenetically highly conserved in many vertebrates, including mammals, fish, amphibians, and birds. Chou-Fasman modeling predicts that this mutation results in a change in the secondary structure of C7 and may affect the activity of the protein. C7 is required for assembly of C5b-9.

We and others have demonstrated that complement components, including the C1q, C3, and the C5b-9 complex, are deposited in the glaucomatous retina and optic nerve head[86], [87]. C1q and C3 are involved in the ‘pruning’ of retinal ganglion cells dendrites during development and have been implicated in glaucomatous damage to the retina and optic nerve[88]. Although the physiological role of complement activation in glaucoma is not entirely understood, studies in a mouse model of ischemic neuropathy indicate that complement inhibition delays retinal ganglion cell degeneration[89]. These findings suggest that complement may specifically target damaged retinal ganglion cells for rapid disruption and removal, possibly to avoid the initiation of an adaptive auto-immune response to retinal antigens as has been described in other diseases[90]. On the other hand, sublytic complement activation may be beneficial for cell survival[91] and it is conceivable that the C5b-9 deposition observed in glaucoma is part of a neuroprotective response of stressed retinal ganglion cells.

In addition to evaluating SNP associations for the primary phenotypes, it is also of interest to identify associations with known disease risk factors, subclasses of disease, and disease complications. For example, central corneal thickness (CCT) has been shown to be a risk factor for glaucoma[2]. In one study eyes with thin corneas (less than 556 microns) have a three-fold greater risk of developing glaucoma than eyes with thick corneas (more than 588 microns). Thus the presence of thin corneas is considered a glaucoma risk factor. In our study, CCT data was available for a subset of patients. Despite the relatively small size of the data set, we observed significant association with CCT at a locus on chromosome 20. Our data indicate that there may be at least one strong genetic locus involved in CCT. It is known that corneal thickness influences intraocular pressure, another risk factor for glaucoma. Thus further study will be required to determine if the chromosome 20 locus is an independent risk locus for glaucoma. An important subclass of glaucoma is normal tension glaucoma. The current study had limited power to detect association for normal tension glaucoma, due to a limited number of patients with this subclass of disease. A major complication of AMD is the presence of CNV. This study identified a suggestive risk locus for CNV. Although there are no reported genes at the associated locus, multiple ESTs map to this region.

## Conclusions

This study reports a genome-wide association study in primary open angle glaucoma. A novel association of POAG to a variation in complement component 7 was identified. In addition, an association of central corneal thickness, a quantitative risk factor for glaucoma, to a SNP in the ribophorin II gene was identified. This study utilized a novel design to maximize the utility of the available genotyping resources and patient cohorts. This design was validated through the observation of AMD associations to the most significant AMD-risk associated alleles – Tyr402His in CFH and Arg69Ser in ARMS2. However, further investigation of this study design is needed to better characterize the power to detect bona fide associations and to correctly reject false associations in comparison to a traditional case-control design. Future work includes focused genotyping in additional large cohorts to further validate the genetic associations.
